# Supplementary material for: DMRforPairs: identifying Differentially Methylated Regions between unique samples using array based methylation profiles
Source: BMC Bioinformatics. 2014 May 15;15:141. doi: 10.1186/1471-2105-15-141 (PMC4046028; doi:10.1186/1471-2105-15-141)
Supplement: Additional file 2 — DMRforPairs output for the comparison of A431-MCF7 and NA17018-NA17105. Please start from the HTML files in each folder. Available via the BMC Bioinformatics website. [file 1471-2105-15-141-S2.zip › 1394847754114233_MOESM2_ESM/A431_MCF7/figures/12621.pdf]

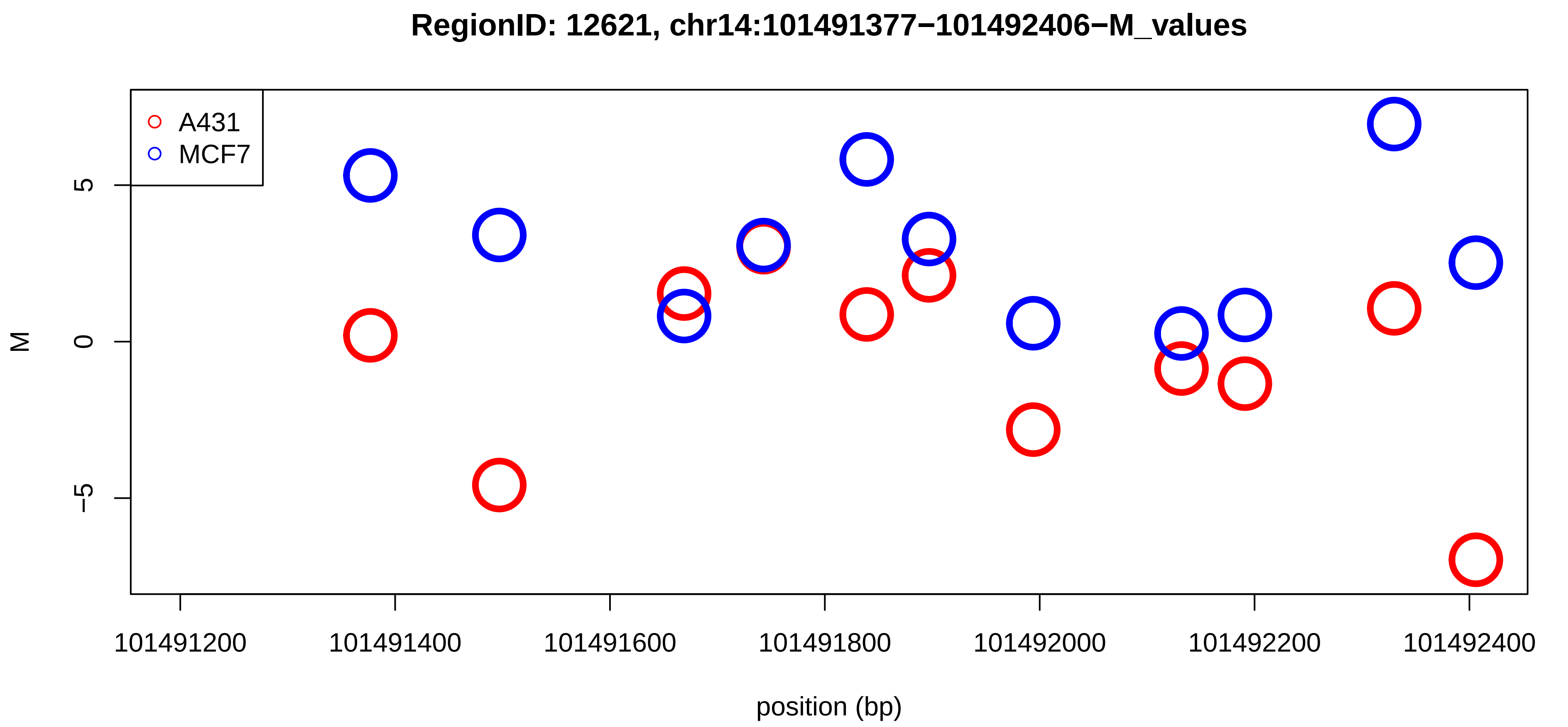
RegionID: 12621, chr14:101491377−101492406−M_values
A431
MCF7
M
5
0
−5
101491200
101491400
101491600
101491800
101492000
101492200
101492400
position (bp)

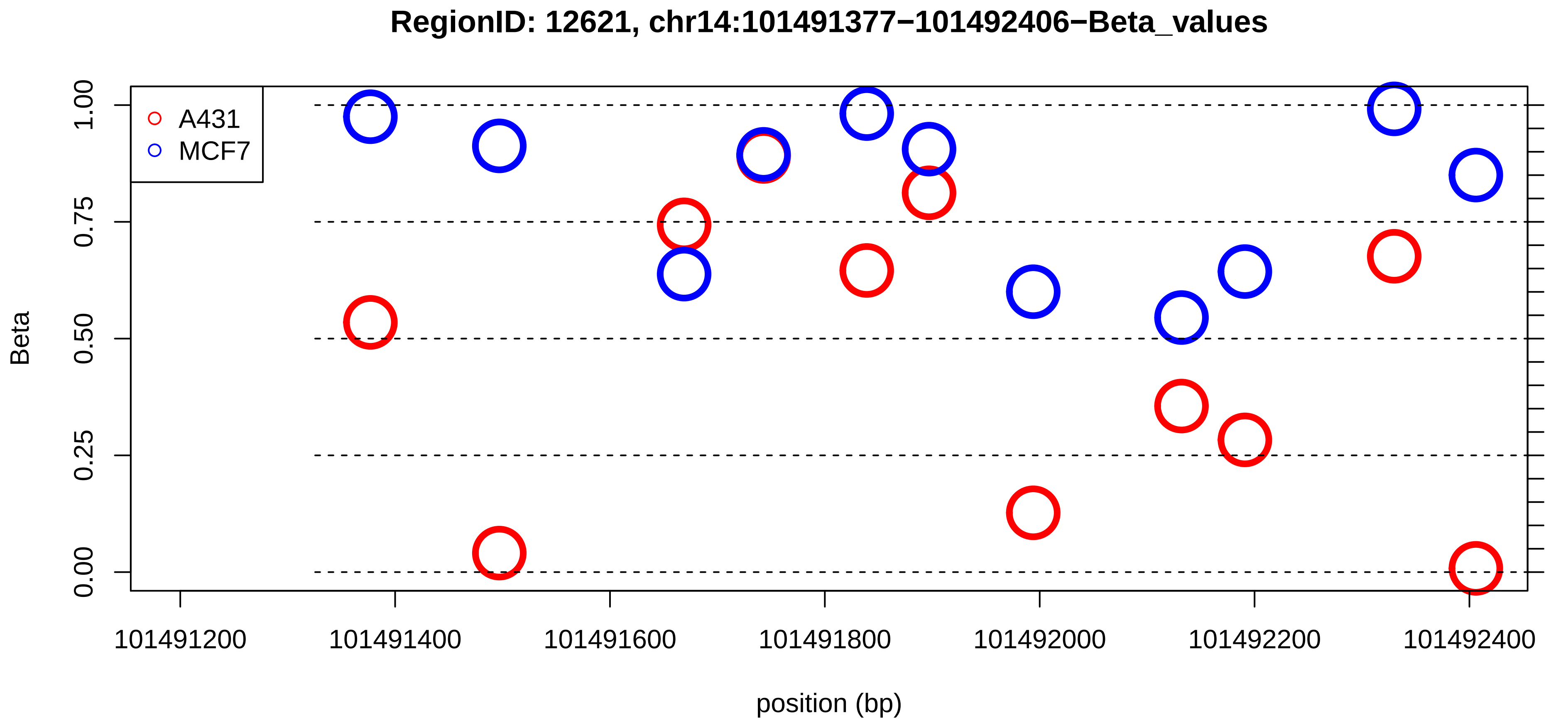
RegionID: 12621, chr14:101491377−101492406−Beta_values
A431
MCF7
Beta
1.00
0.75
0.50
0.25
0.00
101491200
101491400
101491600
101491800
101492000
101492200
101492400
position (bp)

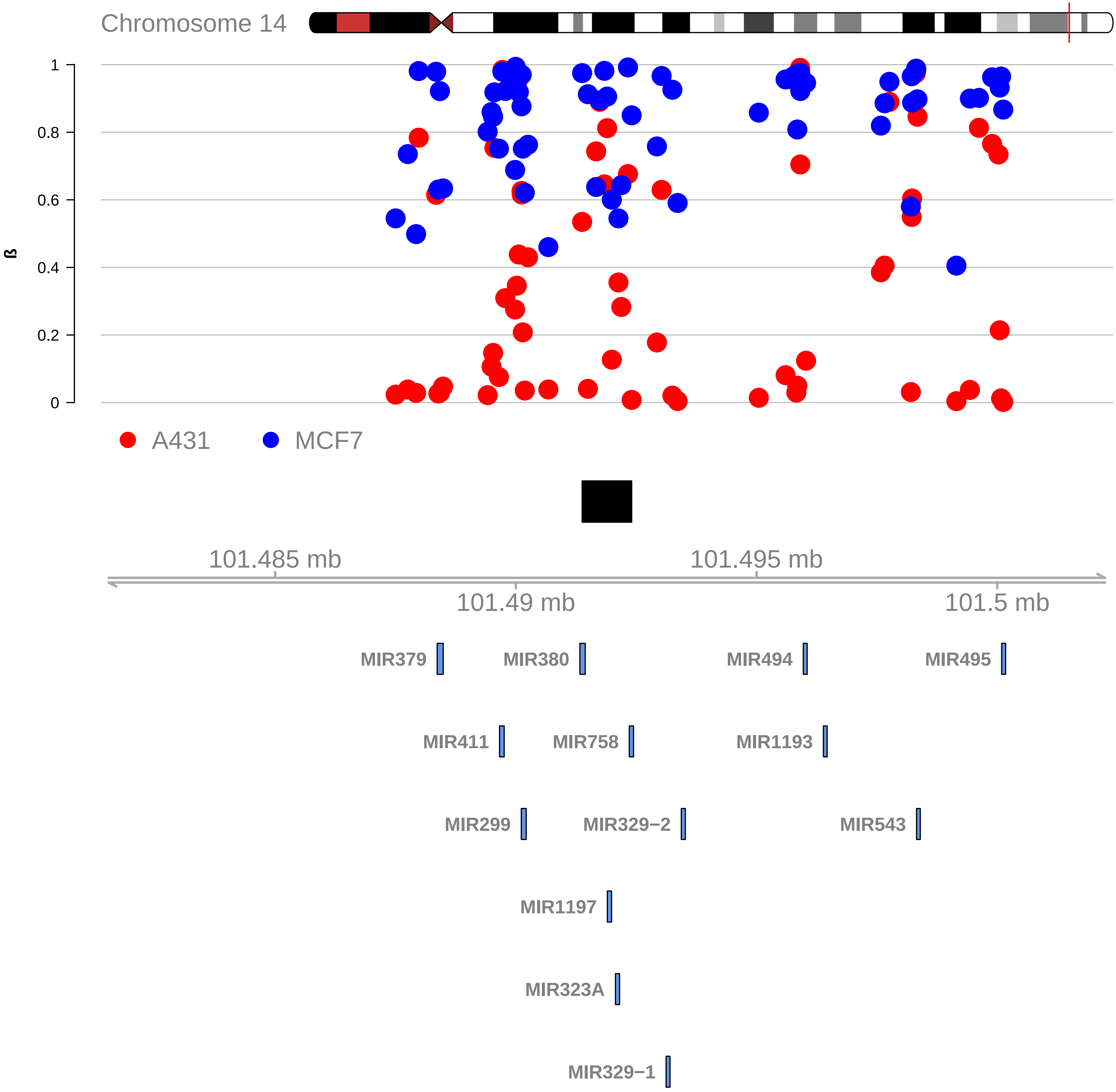

Chromosome 14
1
0.8
0.6
0.4
0.2
0
ß
A431
MCF7
101.485 mb
101.495 mb
101.49 mb
101.5 mb
MIR379
MIR380
MIR494
MIR495
MIR411
MIR758
MIR1193
MIR299
MIR329−2
MIR543
MIR1197
MIR323A
MIR329−1
